# Usefulness of rapid molecular tests in pediatric respiratory tract infections

**DOI:** 10.1186/s13052-022-01200-1

**Published:** 2022-02-03

**Authors:** Simona Nardi, Lorella Carolis, Roberta Iannini, Maria Vittoria De Sandro, Giovanna Solito, Matteo Calafatti, Camilla Gizzi

**Affiliations:** 1 Department of Pediatrics, Sandro Pertini Hospital - ASL RM2, Rome, Italy; 2 Department of Microbiology and Virology, Sandro Pertini Hospital - ASL RM2, Rome, Italy; 3 “Sapienza” University of Rome, Rome, Italy

**Keywords:** Respiratory tract infections, Influenza virus, Respiratory syncytial virus, Rapid diagnostic test, Antibiotic therapy

## Abstract

**Objective:**

To evaluate the role and the advantages of nasopharyngeal swabs in the detection of Influenza A and B viruses and Respiratory syncytial virus through a rapid test based upon a nucleic acid amplification reaction in order to avoid improper antibiotics use.

**Design:**

Case-control retrospective study.

**Setting:**

Emergency Room of “Sandro Pertini” General Hospital, Rome, Italy.

**Participants:**

Children (< 14 years old) who consecutively arrived in the Emergency Department (ED) for respiratory tract infections, without obvious signs of bacterial respiratory tract infections and other comorbidities, in the maximal seasonal incidence period of November-to-March of every year between 2016 and 2020.

**Methods:**

Medical records of children included in the study were retrospectively examined. Children were subdivided according to the following intervals: 2016–2017 and 2017–2018 (Group 1), 2018–2019 and 2019–2020 (Group 2). Children in Group 2 undertook a nasopharyngeal swab, while those in Group 1 did not undergo any specific diagnostic test.

**Primary outcome:**

Avoidance of improper antibiotics administration.

**Results:**

A total of 386 children were included in the study: 174 in Group 1, 212 in Group 2. The Odd Ratio (OR) of prescribing an antibiotic in the groups of children not being swabbed compared to those of children undertaking a swab was 9.21 (CI95% 5.6–15.2, p < 0.001). The overall percentage of hospitalizations, both in the short observation unit or in the pediatric unit, did not differ between the two groups.

**Conclusions:**

Nasopharyngeal swabs for the detection of Influenza A and B viruses and Respiratory syncytial virus proved to be a useful means to a correct and timely diagnosis and allowed for a significant reduction in the prescription of antibiotic therapy.

**Trial registration:**

Retrospectively registered.

## Background

Respiratory tract infections (RTIs) are widespread in the community and commonly occur during the first decade of life. Infants, preschoolers and school-aged children come across, on average, three to ten febrile infections per year [1]. The most common causes of RTIs are viral (e.g., *Rhinovirus, Influenza and Parainfluenza viruses, Respiratory syncytial virus,* etc.) or bacterial (e.g., *Streptococcus pneumoniae, Haemophilus influenzae, Mycoplasma pneumoniae,* etc.). The differential diagnosis between the two infective etiologies (viral vs bacterial) is often challenging as those two share many common symptoms: fever, cough, respiratory distress, pharyngitis, rhinorrhea, earache, headache, swollen regional lymph nodes, malaise and asthenia.

RTIs require careful pediatric evaluation and while the use of antibiotics is justified in the setting of a bacterial infection, the same therapy is not indicated in the presence of a viral infection. Therefore, a correct differential diagnosis is essential to establish an adequate treatment plan.

In the present study we evaluated children who came to our attention in the ED, with respiratory tract infections, without obvious signs of bacterial etiology and without other comorbidities, and compared those who underwent a nasopharyngeal swab for the detection of Influenza A and B viruses (IAV and IBV) and Respiratory syncytial virus (RSV) by means of a rapid test based upon the amplification reaction of nucleic acids, to those who did not. Major end point was to evaluate the role and advantages of this method in making a correct diagnosis and, therefore, planning a suitable therapeutic strategy (with consequent reduction in the overall administration of antibiotics).

## Materials and methods

The Sandro Pertini Hospital in Rome is a general hospital, member of the territorial emergency network as a first level emergency and acceptance department. The catchment area of the hospital includes both the resident population of the district, comprising around 500.000 people, and many non-residents, consisting of around 200.000 people.

The Pediatric Complex Operating Unit belongs to this framework. Its activity covers First Aid services, ordinary hospitalization, nursery, delivery room, Day Hospital, and specialist clinics.

Pediatricians carry out First Aid consultancy activities with direct acceptance of children from 0 to <14 years of age and emergency management, if necessary, in collaboration with other professionals. They also provide consultations to children over the age of 14 up to the age of 18, following the first evaluation carried out by the emergency and acceptance department. The number of accesses to the pediatric Emergency Room in the pre-COVID era was approximately 8000/year, with more than 50% of users displaying characteristics of social fragility (e.g. foreigners, gypsies, low income families, etc.).

The ward welcomes patients with common pediatric pathologies including complex pathologies that require a multidisciplinary approach, carried out in collaboration with the specialists present in the hospital.

For the purpose of our study, we examined the medical records of children (< 14 years old) who consecutively arrived in the Emergency Room of the “Sandro Pertini” General Hospital in Rome (Italy) for RTIs, in the maximal seasonal incidence period of November-to-March of every year between 2016 and 2020. All cases arriving in the emergency room under antibiotic therapy previously prescribed for known or suspected morbidities, children with chronic diseases, those with malformation disorders, and those with obvious signs of bacterial RTIs have been excluded from the study. The signs considered for this last selection were of three different types: clinical and/or instrumental and/or laboratory. More specifically: clinical (otitis, exudative pharingotonsillitis, sinusitis with mucopurulent discharge, lobar pneumonia, lobular bronchopneumonia, pleuritis); instrumental (lung consolidation on Chest Xray); laboratory (complete blood count with white blood cells (WBC) differential, C-Reactive Protein, procalcitonin). Finally among the comorbidities, urinary tract infections were excluded by means of urine dipstick test (if positive for leukocytes and nitrites) and acute infectious gastroenteritis was mostly excluded on a clinical basis, in those children presenting with predominant signs/symptoms of gastroenteritis (i.e. sudden change in stool consistency to loose or watery stools, sudden onset of vomiting, prevalent signs of fluid loss), a positive history of recent contact with someone with acute diarrhoea and/or vomiting, exposure to a known source of enteric infection (possibly contaminated water or food), recent travel abroad. Therefore, our analysis included only infants and children with non-obvious causes of RTIs.

Children have been subdivided according to the following intervals: 2016–2017 and 2017–2018 (Group 1), 2018–2019 and 2019–2020 (Group 2). Children assessed between 2018 and 2020 (Group 2) undertook a nasopharyngeal swab for the detection of IAV and IBV and RSV using a rapid nucleic acid detection test, while those assessed between 2016 and 2018 (Group 1), despite having the same respiratory issues and presenting in the same seasonal period, did not undergo any specific diagnostic test. For statistical purposes, each group was further subdivided according to age (≤12 months or > 12 months). We decided not to consider any patient beyond March 2020 because we believe that their data would have been inevitably conditioned by the events related to the COVID-19 pandemic.

RTI symptoms and their correlated manifestations, considered for the purpose of the analysis, were fever, rhinitis, earache, pharyngitis, cough, lower respiratory tract signs/symptoms (dyspnea, rhonchi, crackles, and wheezes) loss of appetite, vomit, and diarrhea. Loss of appetite, vomit and diarrhea were considered as correlated manifestations only if in association with RTI signs/symptoms. The rate of hospitalization, both in the short observation unit and in the pediatric unit, was also considered. Criteria for hospitalization were: severity of the general condition, fever, age, social fragility, blood tests (complete blood count, procalcitonin, C-Reactive Protein, etc.) and discretion of the caregiver.

Children included in Group 2 have been swabbed using a nasopharyngeal sample collection kit containing a thin swab for elution, to be diluted in 2 ml of sterile physiological solution immediately after collection. The sample workflow consisted in collection, delivery to the microbiology laboratory within one hour from collection, and processing within four hours from arrival to the laboratory. In practical terms, samples were usually delivered to the lab within 15 min from collection and processed within 1 h from delivery. As a consequence, the average waiting time for patients at the ED was 2 h.

For the detection of IAV, IBV and RSV nucleic acids, a rapid molecular method operating on dedicated instrumentation, the ID NOW Instrument (Abbott, California), was used. Every swab was analyzed with three different assays, querying for IAV, IBV and RSV. These assays enable for a multiple automated analysis based upon isothermal nucleic acid amplification technology for the differential and qualitative detection of IAV and IBV nucleic acids, and for the detection of RSV, although the latter assay it is not capable of differentiating between the two antigenic subgroups (A and B) of RSV.

The models, similar to primers, targeting the RNA of influenza A amplify a single region of the PB2 segment, while the models targeting the RNA of influenza B are tailored to a single region of the PA segment. The models targeting the RSV A and B RNAs were used simultaneously, the former amplifying a single region of the non-structural NS2 gene, while the latter being tailored to the nucleocapsid gene N. The molecular signals, identified on a fluorescent basis, were used to specifically identify each target of the amplified RNA.

In general, procedures and therapies have been carried out in accordance with the guidelines proposed by the National Institute for Health and Care Excellence (NICE - www.nice.org.uk) and/or the Italian Society of Pediatrics (https://sip.it – Linee guida). All children, despite the antibiotics therapy and according to individual needs, were treated with Paracetamol and/or Ibuprofen, nasal washes, aerosols with hypertonic solution and/or bronchodilators, corticosteroids and phytotherapic compounds having a soothing effect on cough.

The approval for this study was obtained from the Ethics Committee “Lazio-2”, Rome, Italy (Protocol n. 0107794/2021).

### Statistics

For the purposes of our study, quantitative variables were summarized as mean and standard deviation (SD) or median and Interquartile Range (Q1-Q3). Categorical variables were summarized as number and percentage (%).

Chi Square or Fisher exact test were used to compare categorical variables between two groups and T-Test or Mann-Whitney test were used to compare quantitative data.

Univariable logistic regression was used to evaluate the probability of administering antibiotics with vs without swab and the other several risk factors. A multivariable logistic regression with robust errors, considering factors with *p* value < 0.1 at univariable levels was made to evaluate the adjusted probability to administer antibiotics without swab. Their Odds Ratio (OR) and their confidence Interval at 95% (CI95%) were reported. Stata 16.1 was used for all analyses, and a *p* value < 0.05 was considered statistically significant.

## Results

The two groups did not differ in terms of gender, age, type and number of symptoms or frequency of hospitalization. The symptom of loss of appetite appears to be more frequent in patients who did not get a swab than in those who did (Table 1).

**Table 1:** Baseline characteristics of the included infants

	No swab		Swab		p
N	174		212		
Age in months (mean, SD)	45	40.2	45	36	0.842
Class of age					0.839
< 12 months	68	39.1	85	40.1	
> 12 months	106	60.9	127	59.9	
Sex (n,%)					0.631
Female	77	44.3	99	46.7	
Male	97	55.7	113	53.3	
N symptoms (median, Q1-Q3)	3	3–4	3	3–4	0.623
Symptoms (n,%)					
Fever > 38°	142	81.6	174	82.1	0.906
Rhinitis	95	54.6	124	58.5	0.442
Earache	9	5.2	19	9.0	0.172
Pharyngitis	75	43.1	108	50.9	0.125
Cough	114	65.5	134	63.2	0.638
Lower respiratory tract symptoms	19	10.9	23	10.8	0.999
Loss of appetite	97	55.7	96	45.3	**0.041**
Vomit	26	14.9	22	10.4	0.215
Diarrhea	24	13.8	25	11.8	0.645
Hospitalization (n,%)	137	78.7	155	73.1	0.200

Antibiotic therapy was prescribed to 85% of children not being swabbed and 38% of children who got a swab. The OR of prescribing an antibiotic in the group of children not being swabbed compared to that of children undertaking a swab is 9.21 (CI95% 5.6–15.2, *p* < 0.001).

On multivariable analysis, the probability of prescribing antibiotic therapy to those who did not get a swab remains significantly higher than that for those who did get one, even after adjusting for other variables (number of symptoms, earache, pharyngitis, lower respiratory tract symptoms, loss of appetite, vomiting, diarrhea and hospitalization). Antibiotics were generally prescribed in children presenting with multiple simultaneous symptoms, in presence of pharyngitis, lower respiratory tract symptoms, loss of appetite, vomit, diarrhea and hospitalization. When adjusting for these risk factors, the probability to receive antibiotics remained significantly higher for non-swabbed children, while adjusting for the swab, the probability to take antibiotics remained higher in children with pharyngitis, lower respiratory tract symptoms, vomit, diarrhea and hospitalization (Table 2).

**Table 2:** Univariable and multivariable logistic regression to evaluate the probability of administering antibiotics with vs without swab and the other severalrisk factors

N		No Antibiotic		Antibiotic		Univariable			Multivariable		
		157		229		OR	CI95%	p	OR	CI95%	p
Swab (n,%)											
Yes		131	61.8	81	38.2	1					
No		26	14.9	148	85.1	9.21	5.6-15.2	**<0.001**	29.28	13.5-63.5	**<0.001**
Age in months ( mean, SD)		42	36	47	39	1.00	1.0-1.0	0.266			
Class of age											
<12 months		69	45.1	84	54.9	1					
>12 months		88	37.8	145	62.2	1.4	0.9-2.0	0.152			
Sex (n,%)											
Female		75	42.6	101	57.4	1	0.8-1.7	0.478			
Male		82	39.0	128	61.0	1.0					
N symptoms (median, Q1-Q3)		3	2-4	4	3-5	2.0	1.6-2.5	**<0.001**	0.97	0.7-1.4	0.854
Symptoms (n,%)											
Fever >38°	no	29	41.4	41	58.6	1					
	yes	128	40.5	188	59.5	1.0	0.6-1.8	0.887			
Rhinitis	no	74	44.3	93	55.7	1					
	yes	83	37.9	136	62.1	1.3	0.9-2.0	0.204			
Earache	no	150	41.9	208	58.1	1					
	yes	7	25.0	21	75.0	2.2	0.9-5.2	0.086	4.4	0.9-21.6	0.069
Pharyngitis	no	93	45.8	110	54.2	1					
	yes	64	35.0	119	65.0	1.6	1.0-2.4	**0.031**	2.75	1.5-5.2	**0.002**
Cough	no	56	40.6	82	59.4	1					
	yes	101	40.7	147	59.3	1.0	0.7-1.5	0.978			
Lower respiratory tract symptoms	no	153	44.5	191	55.5	1					
	yes	4	9.5	38	90.5	7.6	2.7-21.8	**<0.001**	16.27	4.0-66.0	**<0.001**
Loss of appetite	no	90	46.6	103	53.4	1					
	yes	67	34.7	126	65.3	1.6	1.1-2.5	**0.017**	1.01	0.5-1.9	0.977
Vomit	no	155	45.9	183	54.1	1					
	yes	2	4.2	46	95.8	19.5	4.7-81.5	**<0.001**	19.71	4.8-81.3	**<0.001**
Diarrhea	no	151	44.8	186	55.2	1					
	yes	6	12.2	43	87.8	5.8	2.4-14.0	**<0.001**	6.29	2.0-20.0	**0.002**
Hospitalization (n,%)	no	72	76.6	22	23.4	1					
	yes	85	29.1	207	70.9	8.0	4.6-13.8	**<0.001**	8.39	3.4-21.0	**<0.001**

**Fig. 1:**
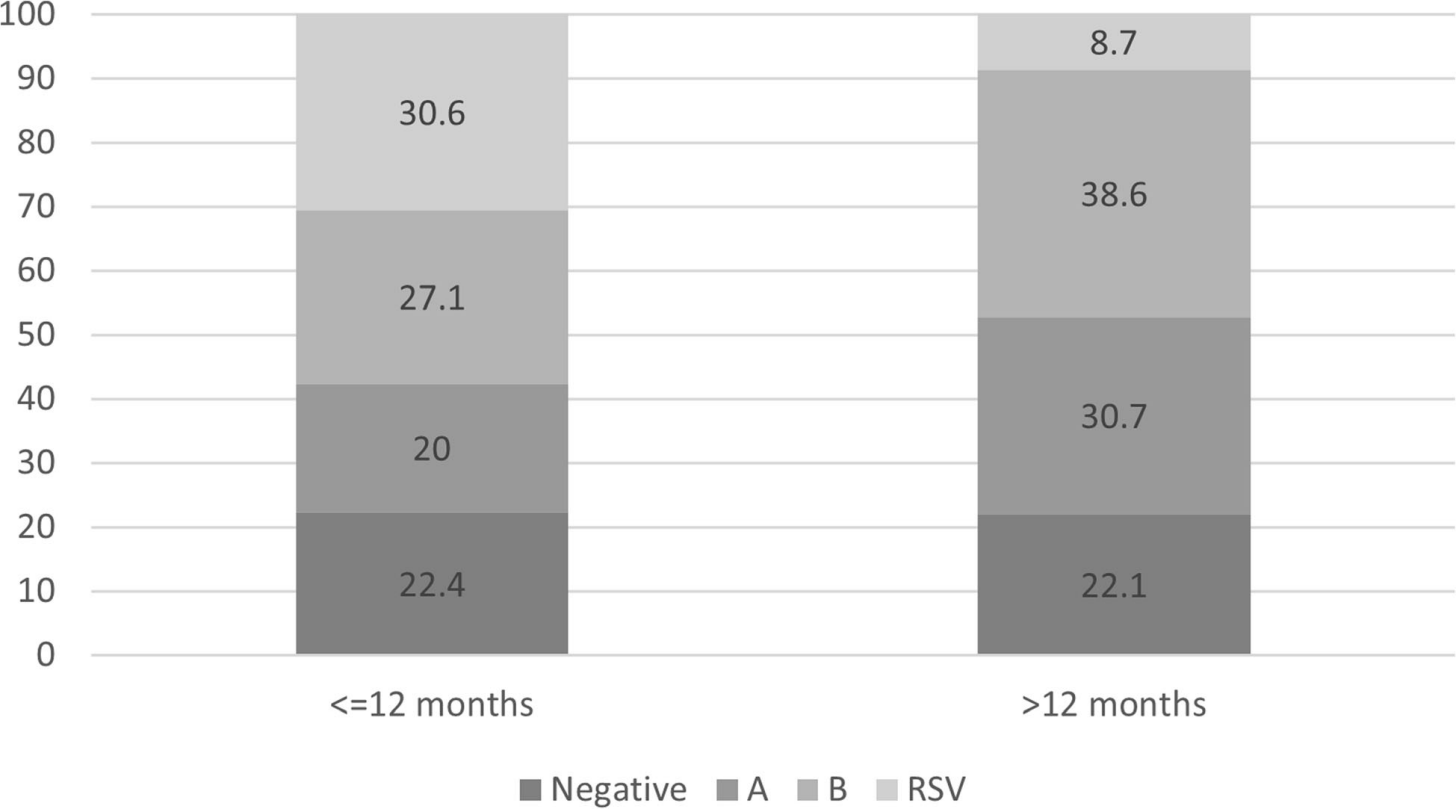
Distribution of nasopharyngeal swab test results according to age

Among the children undertaking a swab, 22% tested negative, 26% tested positive for IAV, 34% positive for IBV and 18% positive for RSV. No difference was observed in the distribution of negative pharyngeal tests according to age. As expected, more children ≤12 months old tested positive for RSV, which was indeed less represented among children > 12 months old (Fig. 1).

In those children who received antibiotic therapy, penicillins (Amoxicillin + Clavulanic Acid) represented our first prescription choice (48%), followed by macrolides (Clarithromycin) (44%). Cephalosporins and aminoglycosides (8%) have been used less frequently, with no major differences between those who got a swab and those who did not.

The overall percentage of hospitalizations, both in the short observation unit or in the pediatric unit, was 78.7% of cases (n.137) in Group 1 and 73.1% (n.155) in Group 2.

## Discussion

The main outcome of our study has been a significant reduction in antibiotics prescription rates among children who undertook a rapid nasopharyngeal test for IAV, IBV and RSV, which allowed for a correct differential diagnosis with respect to bacterial RTIs. In general, the prescription of antibiotics increased with increasing number of symptoms, and was more common in the presence of pharyngitis, lower respiratory tract symptoms, vomiting, diarrhea and hospitalization. It also increased in case of loss of appetite, but this latter result is likely to stem from a disproportion of this symptom between the two groups. As often is very difficult to distinguish between bacterial and viral RTIs, swab molecular testing has been extremely useful in limiting the improper use of antibiotics by distinguishing among the two etiologies. Considering the reality our hospital deals with, the probability of treating an “uncertain” RTI case with antibiotics was 9 times greater when swab molecular testing was not yet available than when it became available. This finding is strengthened by considering that we strictly used the same symptoms and selection criteria to define an infant or child with “non-obvious causes of RTIs” among our study population and that symptoms were equally represented in the two Groups (Table 1). As indicated by the univariable and multivariable logistic regression (Table 2), given the same symptoms, non-swabbed infants were more likely to receive antibiotics.

The respiratory system, despite many specific and non-specific defense mechanisms, is particularly vulnerable to infections, reason why respiratory diseases are among the most commonly encountered in pediatrics. RTIs represent one of the major reasons for seeking care in the pediatric age [1]. The management of these issues touches upon several controversial aspects, and despite the numerous guideline indications, the approach of pediatricians to care is still heterogeneous. The etiological agents involved in RTIs are numerous and often cause reinfections within the same winter season. Among bacteria, commonly encountered pathogens are *Streptococcus pneumoniae, Haemophilus influenzae, Mycoplasma pneumoniae* and *Chlamydia pneumoniae*. Among viruses, the most frequent etiological agents are represented by *IAV* and *IBV* and, especially in infants and young children, by *RSV*. Despite the etiopathology of the infection remains often uncertain, and although antibiotic treatment is not indicated except in high-risk subjects, their use is extremely common in medical practice [1–3]. Penicillin combinations represent the most used class (21.6%), followed by macrolides (12.1%) and cephalosporins (11.8%), in accordance with the guidelines for the treatment of common pediatric infections. It has been hypothesized that as much as 50% of antibiotic prescriptions in children are not actually needed [4]. Consequently, in the recent years, the excessive and sometimes improper use of antibiotics has resulted in the rise of resistant bacterial strains. The issue of antibiotic resistance is being increasingly considered worldwide and requires, according to the WHO, a shift towards a more rational and congruous use of these drugs. This warning holds especially true considering that current research in antibiotic resistance has essentially reached a standstill in the last years [5, 6]. Antibiotic resistance is a growing threat, carrying high costs and serious consequences for the community, such as failure to respond to treatments, prolonged illness and an increased risk of complications [7]. In the European rankings describing the distribution of bacterial resistance across the continent, Italy solidly ranks first along with Greece [5, 8].

Four different types of Influenza viruses have been identified. Of these zoonotic pathogens, only IVA and IVB can spread to people and are hence responsible for seasonal influenza epidemics [9]. One of the characteristics of these viruses is genetic instability. Minor mutations (*genetic drift*) consist of a gradual variation in the sequence of amino acids making up their surface proteins, thus allowing the virus to evade humoral immunity (i.e., antibodies) in previously exposed subjects. These mutations are responsible for seasonal epidemics [10–13]. Bronchiolitis is a seasonal respiratory disease, too. According to the estimates provided by the WHO, it is the most frequent lower RTI in children aged < 1 year and one of the most common causes of hospitalization [14, 15].

It is not often possible to clinically distinguish viral pneumonia (especially if caused by *Adenovirus*) from the disease caused by *Mycoplasma pneumoniae* and other bacterial pathogens [16], yet the differential diagnosis, based on the identification of the possible etiological agent, is of fundamental importance in order to plan a correct therapeutic strategy.

In fact, despite the different etiologies, the phenotypical similarities brought by viral and bacterial infections can make a differential diagnosis quite challenging if that is solely based upon symptomatology. This holds especially true in case of community-acquired pneumonia, where current serological biomarkers are inefficient to specify the etiology of the infection. Indeed, various biomarkers, including C-Reactive Protein, procalcitonin, lipocalin-2 and tumor necrosis factor-related apoptosis-inducing ligand have been evaluated for their ability to differentiate these pneumonia etiologies. For many of these biomarkers, values differ in children with bacterial compared with viral causes of pneumonia, but the reliability of these tests is not sufficiently high to justify routine use. Studies of these biomarkers have also been hampered by the lack of a gold standard to determine pneumonia etiology and the relatively frequent occurrence of viral-bacterial co-infections [16].

Therefore, the detection of viral nucleic acids gives a major contribution to the diagnostic evaluation of children with respiratory infections [1]. Currently, several molecular tests are available for the detection of viral RNA, either relying on conventional RT-PCR (Reverse Transcriptase-Polymerase Chain Reaction) or Real Time RT-PCR methods [17–22]. Those methods are sensitive and specific; however, they are not indicated in every clinical context. On the contrary, the methodology we used of in our study, ID NOW Influenza A and B and ID NOW RSV tests, ensures the differential and qualitative detection of IAV, IBV and RSV from nasal and nasopharyngeal swabs, with many advantages. First, the speed (approximately 15 min) and simplicity of execution is remarkable. Second, the nucleic acid detection can be requested by medical practitioners at any time, during both morning and night shifts, enabling its essential application in emergencies. Third, by not requiring specific technical skills, this test is within reach for virtually any laboratory. Lastly, the sensitivity and specificity of the tests are high. Sensitivity of 96.3% and specificity of 97.4% for IVA, sensitivity of 100% and specificity of 97.1% for IVB and sensitivity of 98.6% and specificity of 98% for VRS [23, 24].

The rapid test is undoubtedly very useful for diagnostics and therapy, but also is in the event that an epidemic outbreaks in confined areas, such as in hospitals, nursing homes and schools. Therefore, it can also serve the purpose of providing epidemiological information to national and international health surveillance authorities.

Our results showed a marked and statistically significant reduction in the number of antibiotic therapy prescriptions in the group of children getting a swab compared to the group not getting any. This result demonstrates how useful can the test be in allowing for a certain diagnosis, and how much, in the absence of the latter, defensive medicine may influence the conduct of a doctor.

From a cost-effectiveness point of view the following considerations can be made. The overall antibiotic prescription rate was 85.0% (148) in Group 1 and 38.2% (81) in Group 2. According to unpublished data taken from our Hospital Farmacy service, the average cost of an oral antibiotic therapy for 7 days is 1.5€ for a child ≤6 years old and 3€ for a child > 6 years old, independently on the type of antibiotic; the average cost of an intramuscular/intravenous antibiotic therapy for 7 days ranges from 3.2€ for aminoglycosides to 3.78€ for cephalosporins and up to 21€ for amoxicillin with clavulanate in children ≤6 years old and from 6.02€ for cephalosporins to 18€ for aminoglycosides and up to 31.5€ for amoxicillin with clavulanate in children > 6 years old. We calculated a total antibiotic cost of 1011€ for Group 1 and of 634€ for Group 2 based upon the age of included patients, antibiotics used and their way of administration. The cost of one rapid test (of the type considered in our study) is 22€, for a total amount of 9.328 € for Group 2.

Concerning the effectiveness of the proposed tests, we could not carry out a thorough benefit analysis in terms of long-term reduction of antibiotic resistance, as it requires complex statistical models and goes beyond the purpose of our manuscript. Yet, it is plausible to assume that a judicious administration of antibiotics can only translate into important economic and health advantages. Economic advantages come from the fact that as resistance develops and more specific antibiotics are needed, their price tend to rise accordingly. Health advantages come from the overall attenuation of antibiotic resistance and the consequent reduction in infections caused by hard-to-treat multi-resistant “bugs”.

All in all, although medical expenses were higher in Group 2, the cost-effectiveness of the rapid tests is high, as the benefits related to the reduction of antibiotic prescriptions greatly outweighs the higher cost of the laboratory tests when compared to the antibiotic therapy itself.

Finally, in a retrospective study like ours, some limitations need to be highlighted. We recognize that the percentage of antibiotic therapy in Group 1 is pretty high and consequently it might be supposed that there were more bacterial infections in this Group. However, as indicated in the Methods section, baseline symptoms were equally distributed in the 2 Groups. In addition, study population selection criteria were strictly similar, making this etiological imbalance between Groups unlikely. We believe that the high antibiotic prescription rate in Group 1 is rather attributable to the subjectivity of the operator, a lack of written protocols at the time of study, and the characteristics of the selected population. The intrinsic diversity of health professionals who have examined the children without following specific protocols and prescribed the therapies evaluated in the study may affect the results: the *modus operandi* of the training schools of origin, the experience and the personal beliefs of every single doctor can inevitably influence the final outcome. Moreover, taking into account the characteristics of our ED population with more than 50% of users displaying social fragility (e.g. foreigners, gypsies, low income families, etc.), a “generous” administration of antibiotics is justified by the difficulties related to children follow-up and the generally low compliance of the families. The same considerations justify the high percentage of hospitalization in both groups. In addition, it should be considered that the population seeking care in our ED generally has a poor cultural background. Lack of general knowledge in medical matter may induce families to reach out to the ED only when their children are already in more compromised conditions.

## Conclusions

The results of this study demonstrate the usefulness of rapid, sensitive and accurate diagnostic tests for the differential diagnosis of RTIs in the pediatric population. The rapid tests based on nasopharyngeal swab allowed not only a correct and timely diagnosis, but also a significant reduction in the use of inappropriate antibiotic therapy.

The tests we used have good sensitivity, and fast execution times. The methodology is simple and feasible for any laboratory.

## Data Availability

The datasets used and/or analysed during the current study are available from the corresponding author on reasonable request.

## Abbreviations

ED: Emergency department
IAV: Influenza A virus
IBV: Influenza B virus
RSV: Respiratory syncytial virus
RTIs: Respiratory tract infections
